# Supplementary material for: Irisin Enhances Mitochondrial Function in Osteoclast Progenitors during Differentiation
Source: Biomedicines. 2023 Dec 14;11(12):3311. doi: 10.3390/biomedicines11123311 (PMC10741766; doi:10.3390/biomedicines11123311)
Supplement: Supplementary file 1 [file biomedicines-11-03311-s001.zip › biomedicines-2505989-supplementary.pdf]

Supplements

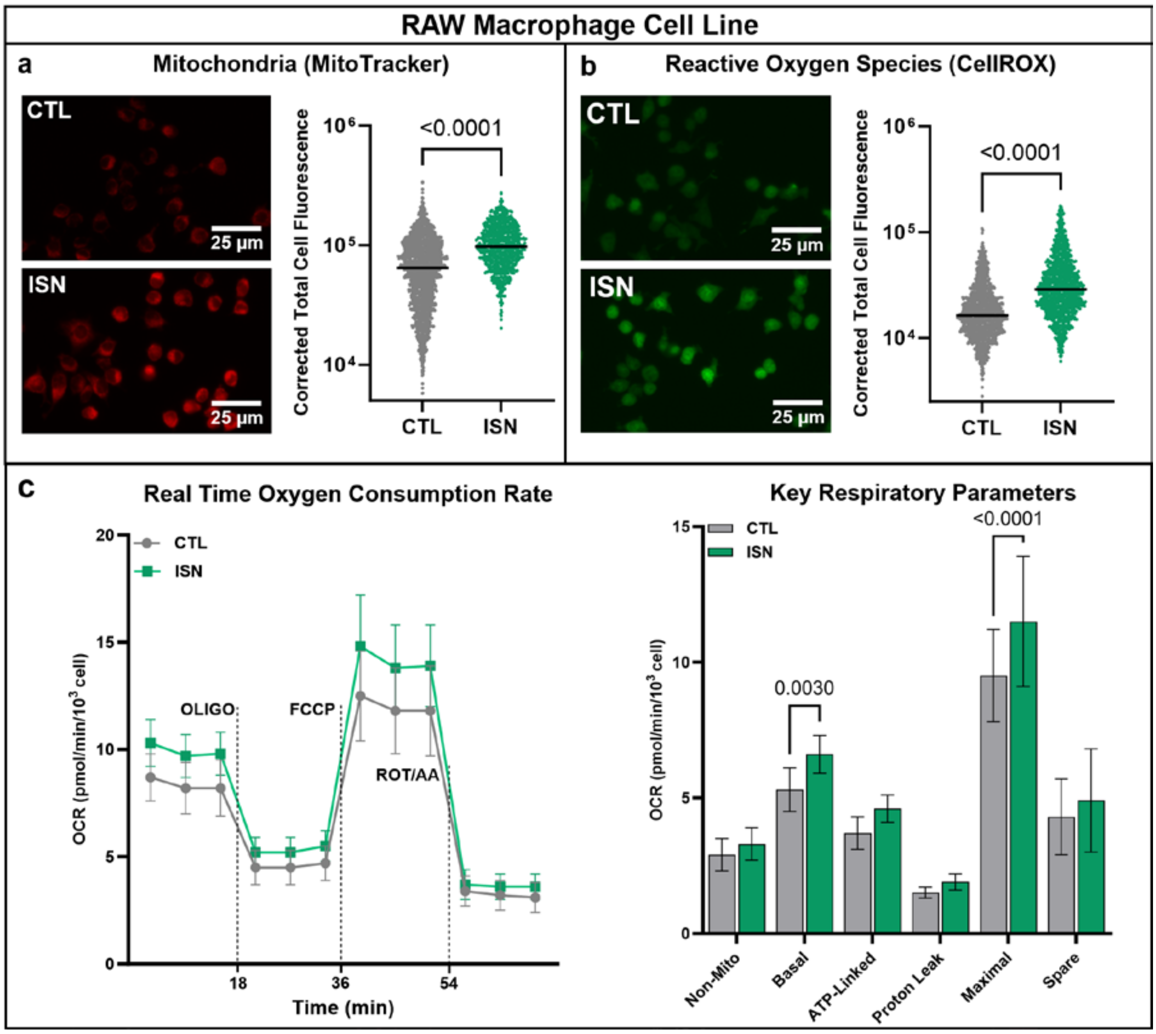

Supplemental Figure S1. Fluorescence imaging and metabolic analysis data from experimental repeats in RAW 264.7 macrophage cell line. Mitochondrial fluorescence via MitoTracker corrected total cell fluorescence significantly increased with 10 ng/mL of irisin (ISN) versus untreated controls (CTL) (a). Reactive oxygen species concentrations via CellROX corrected total cell fluorescence significantly increased with irisin treatment (b). Real-time oxygen consumption rates (OCR) elevated with irisin treatment, leading to increased basal and maximal respiration (c). p-values via unpaired, two-tailed Student's t-test (a,b) and 2-way ANOVA (c), with significant values ($p < 0.05$) are displayed on graph.

Supplemental Table S1. Summary data of technical replicate number (n), mean, standard deviation (StDev), and *p*-value for experimental replicates in primary and RAW cell line osteoclasts (OCL).

Metric	Experiment	Group	n	Mean	StDev	*P*-value
MitoTracker CTCF (Arbitrary Units)	Primary OCL 1	CTL	734	142063	82220	<0.0001
		ISN	509	214165	140989	
	Primary OCL 2	CTL	1341	67641	57318	<0.0001
		ISN	923	81922	71824	
	RAW OCL	CTL	1886	70645	43974	<0.0001
		ISN	859	104107	42427	
CellROX CTCF (Arbitrary Units)	Primary OCL 1	CTL	657	40979	36034	<0.0001
		ISN	758	76617	58977	
	Primary OCL 2	CTL	1200	45144	35614	0.0005
		ISN	626	51034	32040	
	RAW OCL	CTL	1347	20288	13350	<0.0001
		ISN	1231	36735	26948	
Non-Mito OCR (pmol/min/10^3 cells)	Primary OCL 1	CTL	23	1.5	0.5	0.9842
		ISN	23	1.7	0.5	
	Primary OCL 2	CTL	16	2.4	0.3	0.9999
		ISN	16	2.5	0.4	
	RAW OCL	CTL	20	2.9	0.6	0.8585
		ISN	22	3.3	0.6	
Basal OCR (pmol/min/103 cells)	Primary OCL 1	CTL	23	3.1	0.9	0.8931
		ISN	23	3.4	0.7	
	Primary OCL 2	CTL	16	4.6	0.8	0.9565
		ISN	16	4.9	0.5	
	RAW OCL	CTL	20	5.3	0.8	0.003
		ISN	22	6.6	0.7	
ATP-Linked OCR (pmol/min/103 cells)	Primary OCL 1	CTL	23	1.9	0.7	0.9842
		ISN	23	2.1	0.5	
	Primary OCL 2	CTL	16	3.1	0.6	0.9945
		ISN	16	3.3	0.3	
	RAW OCL	CTL	20	3.7	0.6	0.0877
		ISN	22	4.6	0.5	
Proton Leak OCR (pmol/min/103 cells)	Primary OCL 1	CTL	23	1.2	0.2	0.9997
		ISN	23	1.3	0.3	
	Primary OCL 2	CTL	16	1.5	0.3	0.9999
		ISN	16	1.6	0.3	
	RAW OCL	CTL	20	1.5	0.2	0.8585
		ISN	22	1.9	0.3	
Maximal OCR (pmol/min/103 cells)	Primary OCL 1	CTL	23	8.5	1.6	<0.0001
		ISN	23	10.9	1.7	
	Primary OCL 2	CTL	16	10.2	1.8	0.0064
		ISN	16	11.4	1.8	
	RAW OCL	CTL	20	9.5	1.7	<0.0001
		ISN	22	11.5	2.4	
Spare OCR (pmol/min/103 cells)	Primary OCL 1	CTL	23	5.4	1.3	<0.0001
		ISN	23	7.5	1.5	
	Primary OCL 2	CTL	16	5.6	1.3	0.0785
		ISN	16	6.5	1.6	
	RAW OCL	CTL	20	4.3	1.4	0.4837
		ISN	22	4.9	1.9	
